# Effect of Thyrotropin on Osteopontin, Integrin α_v_β_3,_ and VCAM-1 in the Endothelium via Activation of Akt

**DOI:** 10.3390/ijms17091484

**Published:** 2016-09-20

**Authors:** Yumeng Yan, Fengwei Jiang, Yaxin Lai, Haoyu Wang, Aihua Liu, Chuyuan Wang, Yuanyuan Zhang, Weiping Teng, Zhongyan Shan

**Affiliations:** Department of Endocrinology and Metabolism, Institute of Endocrinology, Liaoning Provincial Key Laboratory of Endocrine Diseases, The First Affiliated Hospital of China Medical University, China Medical University, No. 155 Nanjing North Street, Shenyang 110001, Liaoning, China; cherylyanyumeng@hotmail.com (Y.Y.); winnterrain0923@tom.com (F.J.); laiyaxin811005@126.com (Y.L.); why_endocrinology@foxmail.com (H.W.); liuaihuacmu@163.com (A.L.); chuyuan0215@163.com (C.W.); yuan-anpo@126.com (Y.Z.); twp@vip.163.com (W.T.)

**Keywords:** thyrotropin, atherosclerosis, subclinical hypothyroidism, osteopontin

## Abstract

Numerous epidemiological studies have shown that subclinical hypothyroidism (SCH) can impair endothelial function and cause dyslipidemia. Studies have evaluated the effects of thyroid stimulating hormone (TSH) on endothelial cells, but the mechanism underlying the proatherosclerotic effect of increased TSH levels remains unclear. In the present study, SCH rat models were established in thyroidectomized Wistar rats that were given l-T_4_ daily. The results showed that in vivo, the expression of osteopontin (OPN) vascular cell adhesion molecule (VCAM-1), and levels of integrin α_v_β_3_ in the aortic tissue in SCH and Hypothyroidism (CH) groups was higher than in the control group. However, the effect in the SCH group was higher than in the CH group. In vitro, results showed that different concentration and time gradients of TSH stimulation could increase the expression of OPN, VCAM-1, and integrin α_v_β_3_, and this was accompanied by extracellular signal regulated kinase 1/2 (Erk1/2) and Akt activation in human umbilical vein endothelial cells (HUVECs). TSH induced elevation of these proatherosclerotic factors was partially suppressed by a specific Akt inhibitor but not by a specific Erk inhibitor. Findings suggested that the endothelial dysfunction caused by SCH was related to increased proatherosclerotic factors induced by TSH via Akt activation.

## 1. Introduction

Subclinical hypothyroidism (SCH) which is defined as elevated serum thyroid stimulating hormone (TSH) levels with concomitant normal serum free thyroid hormone levels (free T3 (fT3) and free T4 (fT4)), has garnered more attention in recent years. The morbidity SCH is 4%–20% of the adult population [1,2]. Numerous epidemiological studies demonstrated that SCH could be a strong risk indicator for atherosclerosis [3] and is associated with increased carotid intima-media thickness (IMT) [4] and dyslipidemia [5,6,7].

Osteopontin (OPN), which is a phosphorylated glycoprotein originally found in bone, has recently been shown to be involved in the pathological processes of which atherosclerosis is a key factor [8,9]. Numerous articles report increased OPN expression in human atherosclerotic plaques and its association with the severity of coronary artery disease [10,11]. Integrin α_v_β_3_, which acts as an adhesion receptor, also plays a crucial role in the progression of atherosclerosis. Previous studies have demonstrated that both the endothelium along the lumen of nonatherosclerotic diffuse intimal thickening and atherosclerotic plaques are associated with increased expression of integrin α_v_β_3_ [12,13]. Vascular cell adhesion molecule (VCAM-1) is an important adhesion molecule for endothelial cells and also plays a critical role in inflammation, such as mediating the interaction of monocytes with the arterial wall.

Vascular endothelial cells, such as human aorta endothelial cells and human umbilical vein endothelial cells (HUVECs), express TSH receptor (TSHR) [14,15]. Endothelial dysfunction is considered a crucial early step in the pathological process of atherosclerosis. Some studies have provided evidence for the acute enhancement of recombinant human thyrotropin (rhTSH) in the endothelium-mediated vasodilation independent of thyroid hormones [16,17], but other studies have suggested the opposite [18]. Studies performed on different types of endothelial cells have shown that TSH stimulation can rapidly elevate cyclic adenosine monophosphate (cAMP) concentration, accompanied by increased nitric oxide (NO) and decreased vasoconstrictor endothelin, which proved that TSH had a protective effect on endothelial function [14]. Nevertheless, some studies have demonstrated that TSH can induce lower levels of endothelial nitric oxide synthase (eNOS) and prostaglandin I_2_ (PGI_2_) and higher levels of endothelin and plasminogen activator inhibitor-1 (PAI-1) expression. This was consistent with the destructive effect of TSH [15]. However, it remains unclear whether TSH has any direct impact on proatherosclerotic factors, such as OPN, VCAM-1, and integrin α_v_β_3_, and its signaling pathway remains to be elucidated.

In the present study, the effect of TSH on OPN, VCAM-1, and integrin α_v_β_3_ was determined in HUVECs, as well as its possible mechanisms. The results also showed the proatherosclerotic effect of subclinical hypothyroidism in Wistar rat models.

## 2. Results

### 2.1. Hormone Levels in Rats of Three Groups of Rats

The Wistar rats were randomly divided into three groups, control (CON), subclinical hypothyroidism (SCH), and hypothyroidism (CH) groups. To confirm the thyroid status of each group, plasma levels of total thyroxine (TT_4_) and TSH were detected as shown in Table 1. The CH group showed significantly higher TSH levels and lower TT4 levels than did in the CON group. Then, 4 or 14 weeks after l-T_4_ injection, the SCH group showed significantly higher TSH levels than the CON group, but there was no statistically significant difference in TT4 between the SCH and CON groups. In summary, the CH and SCH rat models were successful.

### 2.2. Western Blot Analysis of Osteopontin (OPN), Integrin α_v_β_3_, and Vascular Cell Adhesion Molecule (VCAM-1) Expression of Aorta Tissues from CH, SCH, and CON Groups

**Figure 1:**
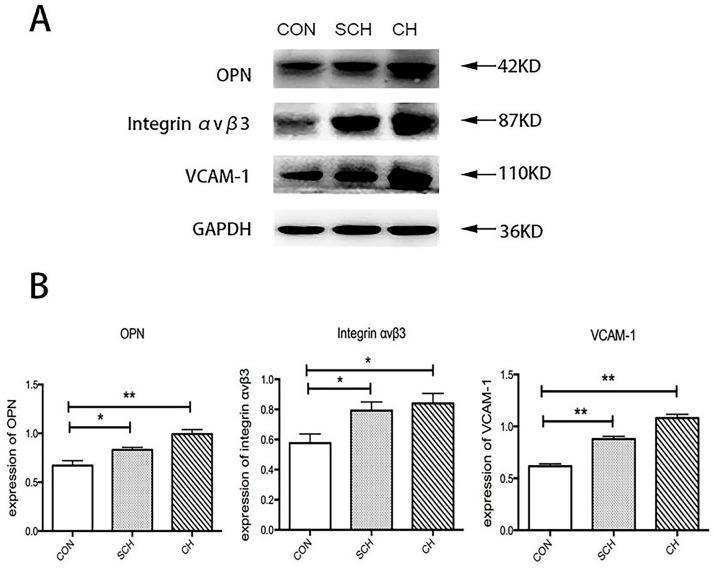
Osteopontin (OPN), integrin α_v_β_3_, and vascular cell adhesion molecule (VCAM-1) expression in aorta; tissues of control (CON), subclinical hypothyroidism (SCH), and hyoithyroidism (CH) rats. (**A**) The bands depict representative findings regarding protein expression levels of OPN, integrin α_v_β_3_, and VCAM-1 protein expression in the aortic tissues in CON, SCH, and CH rats. These were evaluated by Western blotting using protein extracted from 20 mg of aorta tissues; (**B**) The bar graph shows the results of the semiquantitative measurements of OPN, integrin α_v_β_3_, and VCAM-1. Values are shown as the means ± SEM. * *p* < 0.05 versus CON group; ** *p* < 0.01 versus CON group.

The Western blot results are shown in Figure 1. OPN, integrin α_v_β_3_, VCAM-1, and Glyceraldehyde-3-phosphate dehydrogenase (GAPDH) levels were measured at 42, 87, 110, and 36 KD, respectively. The expression of OPN, integrin α_v_β_3_, and VCAM-1 was significantly higher in aorta tissues from the SCH group than in those from the CON group, and the expression of OPN, integrin α_v_β_3_, and VCAM-1 in aorta tissues was also significantly higher in the CH group. Although the expression of OPN, integrin α_v_β_3_, and VCAM-1 was higher in aorta tissues from the CH group than in those from the SCH group, this difference was not statistically significant.

### 2.3. Immunohistochemical Analysis of OPN, Integrin α_v_β_3_, and VCAM-1 Expression of Aorta Endothelium from CH, SCH, and CON Groups

**Figure 2:**
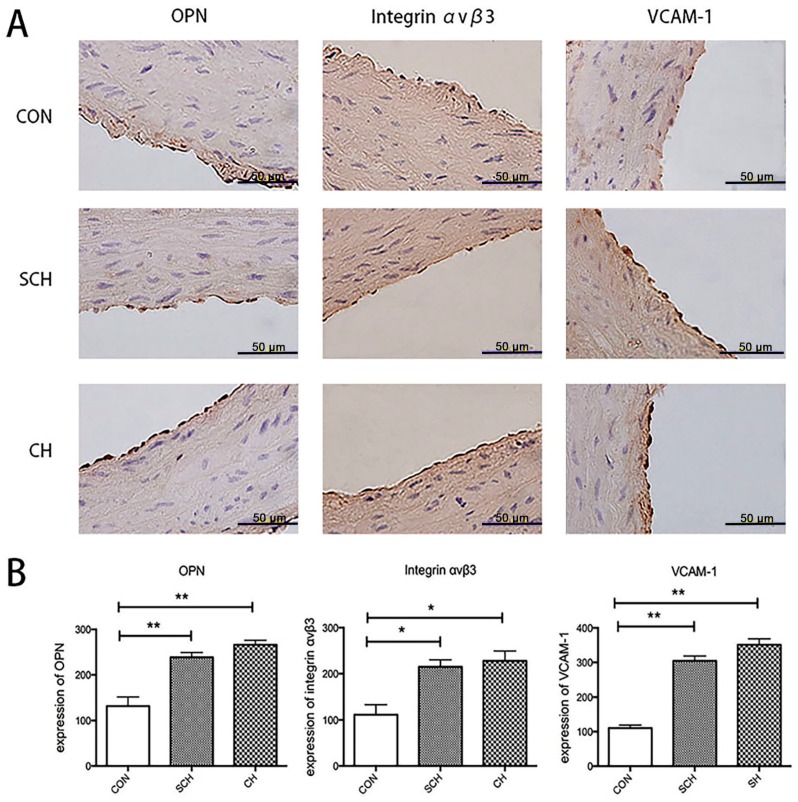
OPN, integrin α_v_β_3_, and VCAM-1 expression in the aorta endothelium from CON, SCH, and CH rats, as indicated by immunostaining. (**A**) Immunostaining of OPN, integrin α_v_β_3_ and VCAM-1 in the endothelia of aortas from CON, SCH, and CH rats; (**B**) Semiquantitative analysis of the difference in OPN, integrin α_v_β_3_, and VCAM-1 expression in the endothelia of the aortas from CON, SCH, and CH rats. Data were presented as the means ± SEM. * *p* < 0.05 versus CON group; ** *p* < 0.01 versus CON group. Scale bar = 50 µm.

The results of the immunohistochemical analysis are shown in Figure 2. After immunohistochemical staining, positive endothelial cells appeared yellow-brown. In the CON aorta group, few endothelial cells in the endothelium were found to be positive for OPN, integrin α_v_β_3_, or VCAM-1. However, the SCH and CH aorta groups showed significantly more cells that were positive for OPN, integrin α_v_β_3_-positive, and VCAM-1. The optical density (OD) of positive cells in each field of the slide was determined to evaluate the average OD. The OD values of OPN-positive, integrin α_v_β_3_-positive, and VCAM-1-positive endothelial cells from the SCH and CH groups were statistically significantly higher than those of the CON group (Figure 2B). Although the OD values of the CH group were much higher than those of the SCH group, the difference was not statistically significant, which is consistent with the results of the Western blot analysis.

### 2.4. Morphological Changes in the Aortic Endothelium in CH, SCH, and CON Groups by TEM

**Figure 3:**
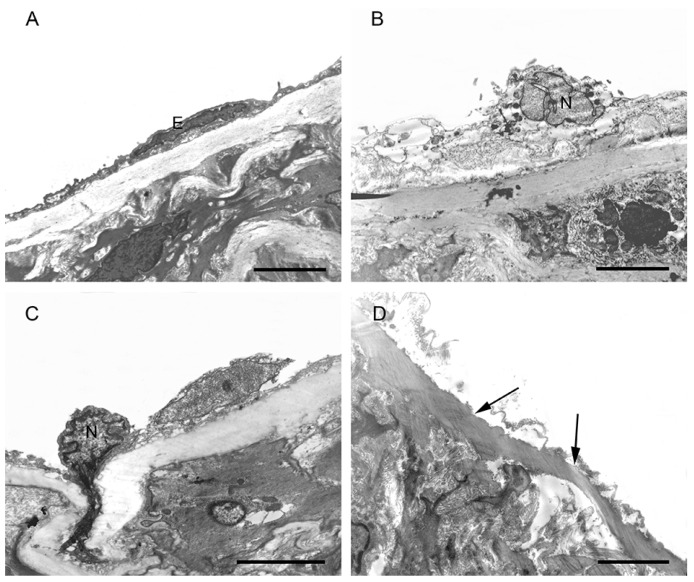
The figure shows the endothelium in the CON, SCH, and CH groups. (**A**) Normal endothelial cell (E) in the CON group; (**B**) Dissolved endothelial cell membrane and abnormal nuclear (N) feature in the SCH group; (**C**) Dissolved endothelial cell membrane and heterogeneous chromatin edge accumulation in the CH group; (**D**) Endothelial cells shed and parts of the elastic membrane exposed in the CH group (→). Scale bar = 4 µm.

Under TEM, the endothelial cells of the aorta endothelium from the CON group showed complete structures which included clear nuclear and cell membranes, and there were tight junctions between the endothelial cells (Figure 3A). In the SCH group, some of the endothelial cells of the aortic endothelium had broken and dissolved cell membranes, and abnormal nuclear features; some mitochondria showed vague outer membranes and crest degeneration not observed in the CON group (Figure 3B). In the CH group, some endothelial cells had been shed and parts of the elastic membrane were exposed, some of the nuclear features were abnormal, and some cells showed heterogeneous chromatin edge accumulation, dissolution of the cell membrane, and even some degrees of breakage. In the cytoplasm, some of the cells showed mitochondrial vacuolar degeneration and broken outer membranes not observed in the CON group (Figure 3C,D).

### 2.5. Effects of TSH on OPN, Integrin α_v_β_3_, and VCAM-1 in Human Umbilical Vein Endothelial Cells (HUVECs)

**Figure 4:**
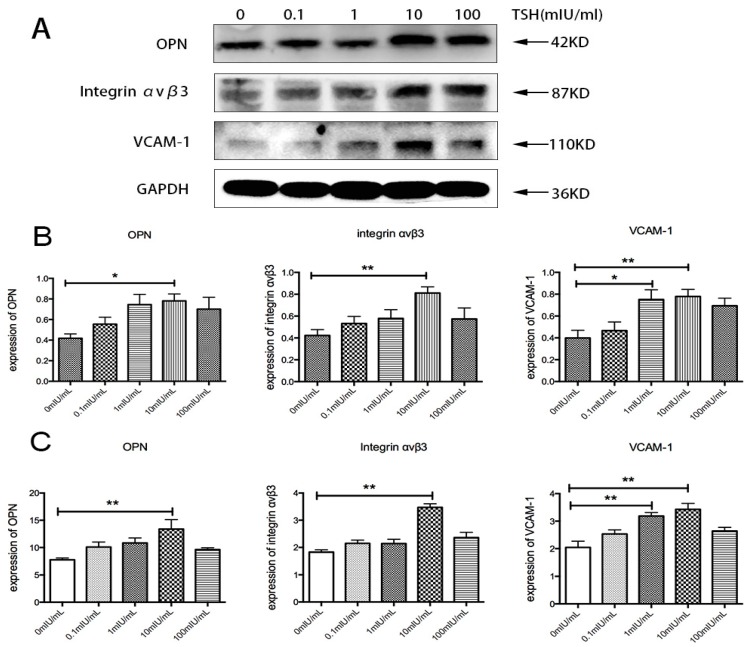
Effects of concentrations of TSH (0, 0.1, 1, 10, 100 mIU/mL) in HUVECs over 24 h. (**A**) The bands depict representative findings of OPN, integrin α_v_β_3_, and VCAM-1 protein expression levels in HUVECs stimulated by different concentration of TSH; (**B**) The bar graphs showed the results of the semiquantitative measurements of OPN, integrin α_v_β_3_, and VCAM-1, respectively; (**C**) The bar graphs showed the results of the quantitative analysis of OPN, integrin α_v_β_3_, and VCAM-1 from real-time PCR. The OPN, integrin α_v_β_3_, and VCAM-1 mRNA levels were expressed as ratios relative to GAPDH. The results are shown as the means ± SEM of three independent experiments. * *p* < 0.05 versus control; ** *p* < 0.01 versus control.

**Figure 5:**
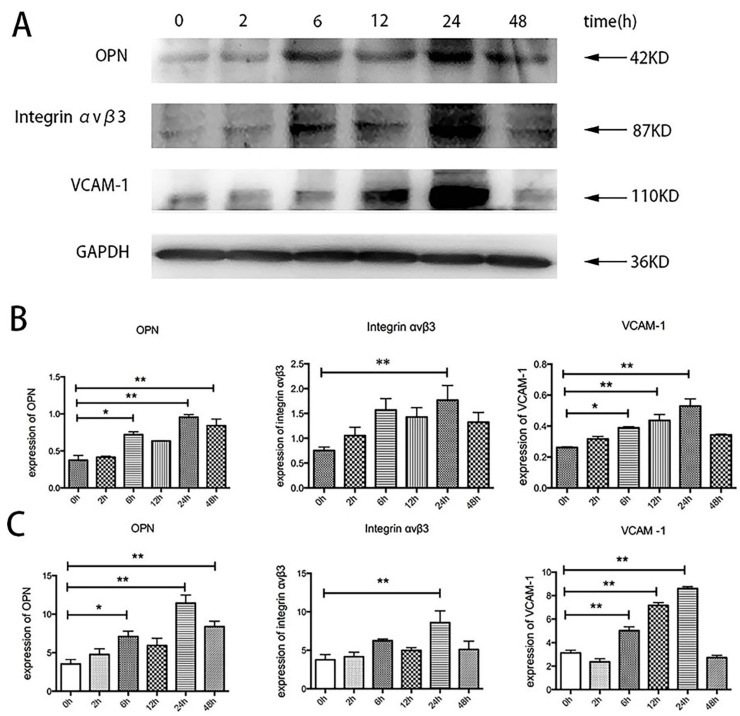
The effects of 10 mIU/mL TSH for 0, 2, 6, 12, 24, and 48 h in HUVECs. (**A**) The bands depicted representative protein expression level findings for OPN, integrin α_v_β_3_, and VCAM-1 in HUVECs stimulated by different durations of TSH; (**B**) The bar graphs show the results of the semiquantitative measurements of OPN, integrin α_v_β_3_, and VCAM-1; (**C**) The bar graphs show the results of the quantitative analysis of OPN, integrin α_v_β_3_, and VCAM-1 from real-time PCR. The OPN, integrin α_v_β_3_, and VCAM-1 mRNA levels are expressed as ratios relative to GAPDH. The results are shown as the means ± SEM of three independent experiments. * *p* < 0.05 versus control; ** *p* < 0.01 versus control.

To investigate whether TSH could directly induce a proinflammatory effect on HUVECs, the expression of OPN, integrin α_v_β_3_, and VCAM-1 was detected after HUVEC stimulation with TSH. HUVECs were starved in serum-free ECM for 12h and then treated with various concentrations of TSH (0, 0.1, 1, 10, 100 mIU/mL) for 24 h. As shown in Figure 4, TSH increased OPN expression in a dose-dependent manner from 0.1 to 10 mIU/mL, and the increase became significant after treatment with 10 mIU/mL TSH. With 10 mIU/mL TSH, the expression of OPN increased by 1.87-fold compared with the controls. As shown in Figure 5, the increase in OPN was evident at 6, 24, and 48 h after treatment with 10 mIU/mL TSH and with a more significant effect at 24 h. The expression of OPN at 6, 24, and 48 h increased by 1.92-, 2.55-, and 2.24-fold, respectively compared with the controls. The results of the Western blot were consistent with Real Time PCR.

The effect of TSH on integrin α_v_β_3_ was also directly investigated. The upregulation of integrin α_v_β_3_ was statistically significant at 24 h, and it was 2.35-fold that of the controls (Figure 4). The results also showed that the effect of TSH on integrin α_v_β_3_ was similar to the effect on OPN. As shown in Figure 4, 10 mIU/mL TSH stimulation statistically significantly upregulated integrin α_v_β_3_ at 6, 24, and 48 h, and the increase was 2.08-, 2.35-, and 1.76-fold, respectively, that of the controls.

The results indicated that various concentrations could increase VCAM-1 expression, and the upregulation was statistically significant at 1 and 10 mIU/mL TSH for 24 h; the increase was 1.88- and 1.95-fold that of the controls, respectively, as shown in Figure 4. When treated with 10 mIU/mL TSH for 0, 2, 6, 12, 24, and 48 h, the increase in VCAM-1 expression became evident at 6, 12, and 24 h and was 1.49-, 1.67-, and 2.02-fold, respectively, of that of the controls (Figure 5). The expression of VCAM-1 protein after TSH stimulation was similar to mRNA detected by RT-PCR.

### 2.6. Effects of Specific Inhibitors on TSH-Induced OPN, Integrin α_v_β_3_, and VCAM-1 Upregulation in HUVECs

**Figure 6:**
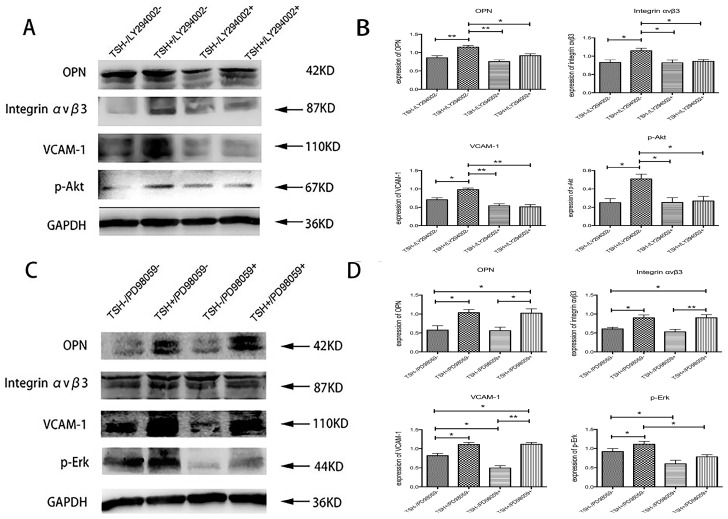
Effects of specific inhibitors on TSH-induced OPN, integrin α_v_β_3_, and VCAM-1 upregulation in HUVECs. HUVECs were pretreated with either 20 µmol/L LY294002 or 20 µmol/L PD98059 for 1 h and then treated with 10 mIU/mL TSH for 24 h. (**A**) The bands depicted representative findings for the protein expression levels of p-Akt, OPN, integrin α_v_β_3_, and VCAM-1 in HUVECs as determined by Western blotting; (**B**) The bar graphs show the results of the semiquantitative measurements of of p-Akt, OPN, integrin α_v_β_3_, and VCAM-1; (**C**) The bands depicted representative findings for protein expression levels of p-Erk, OPN, integrin α_v_β_3_, and VCAM-1 in HUVECs, as determined by Western blotting; (**D**) The bar graphs showed the results of the semiquantitative measurements of p-Erk, OPN, integrin α_v_β_3_, and VCAM-1. The results are shown as the means ± SEM of three independent experiments. * *p* < 0.05 versus control; ** *p* < 0.01 versus control.

TSH was found to upregulate the activation of both p-Akt and p-Erk. The specific Akt inhibitor LY294002 prevented TSH-induced upregulation of OPN, integrin α_v_β_3_, and VCAM-1 to a significant extent (Figure 6A,B). The specific Erk inhibitor PD98059 showed no effect on basal or TSH-induced OPN, integrin α_v_β_3_, or VCAM-1 expression (Figure 6C,D).

## 3. Discussion

Endothelial cells from healthy arteries exhibit a non-adhesive and anti-thrombotic state which is crucial to cardiovascular control in response to substances given off by nerves, circulating hormones (e.g., thyroid hormones, TSH), and other stimulants [19]. In this way, endothelial function plays specific and critical roles in the pathogenesis of atherosclerosis. Its agents include relaxing and contracting factors, such as nitric oxide (NO) and endothelin (ET), and pro-inflammatory and anti-inflammatory mediators [20]. Injured endothelia induce atherosclerosis by inducing smooth muscle cell migration and proliferation, increasing expression of growth factors, and disrupting the balance of the coagulation and fibrinolytic system [21]. Some studies have shown subclinical hypothyroidism to be associated with increased carotid intima-media thickness (IMT), a significant marker of the early stage of atherosclerosis [4,22,23]. The association between subclinical hypothyroidism and atherosclerosis could be complex. It might be attributable to dyslipidemia, hypertension, metabolic disorders, and increased level of serum TSH.

To assess the long-term effects of SCH on the cardiovascular system, SCH and CH models were established in Wistar rats to demonstrate the proatherosclerotic effects on aorta tissues. l-T_4_ treatment was conducted long-term, and the levels of thyroid hormones and TSH were assessed for 14 weeks. Some of the endothelial cells in the SCH and CH groups showed irregular cell membranes, abnormal nuclear features, and abnormal mitochondria, as detected by TEM, which were not observed in the CON group. The results suggested that the integrity of the endothelium was broken, which might lead to the expression of some adhesion molecules. This was consistent with previous studies that showed subclinical hypothyroidism and hypothyroidism to be associated with the early stages of atherosclerosis [3,22,24,25].

OPN is a multifunctional extracellular matrix protein. It can be biosynthesized by several types of cells, such as osteoblasts, smooth muscle cells, macrophages, and endothelial cells. Its crucial role in the pathological process of atherosclerosis and coronary artery disease occurs through its ability to induce chemotactic movement [10,26]. OPN has also been associated with thyroid disease due to its pro-inflammatory effect [27,28]. In propylthiouracil-induced (PTU) CH rat models, OPN expression was found to be higher in the aorta and heart. Furthermore, supplementation with T_3_ was found to decrease serum lipid levels and reduce OPN mRNA expression in the aorta [29]. Consistent with this result, the current study also showed significantly higher levels of OPN in the aorta tissues in the CH and SCH groups compared to the CON group. In vitro, treatment with TSH from 0.1 to 10 mIU/mL for 24 h elevated the mRNA and protein expression of OPN in a dose-dependent manner in HUVECs, which may have caused the lower levels of OPN expression observed in SCH aortic tissues relative to the CH group. The results of immunohistochemical analysis of OPN also showed more expression in the endothelium in the CH and SCH groups. Moreover, the direct effect of TSH on OPN in vitro also indicated that TSH might have an independent influence on the lumen of the aorta.

Studies in animal models of vascular injury showed that factors that promote and restore endothelial integrity are of great importance. Integrins and their ligands, which are cell-cell and cell-matrix adhesive proteins, play a crucial role in protecting endothelial integrity. One receptor for OPN, integrin α_v_β_3_, has been shown to be an important migratory receptor in endothelial cells and participates in several pathological processes, such as vascular remodeling [30] and angiogenesis [31]. Previous studies have demonstrated that both the endothelium along the lumen of nonatherosclerotic diffuse intimal thickening and atherosclerotic plaques express more integrin α_v_β_3_ than do normal vessels [12,13]. Carotid injury in aopE**^−^**^/**−**^ mice induced neointima and media thickening, and the expression of integrin α_v_β_3_ was upregulated in the vessel wall [32]. In the present study, integrin α_v_β_3_ expression was significantly increased in aorta tissues in both the SCH and CH groups, as indicated by immunochemistry, which could suggest that SCH and CH rats had abnormal endothelia. The expression of integrin α_v_β_3_ was higher in aorta tissues from CH than in the SCH group; there were no statistically significant differences between the two groups. No obvious morphological changes were observed in the endothelium by hematoxylin and eosin (HE) staining. This might be attributable to the duration of the experiment which was not sufficiently long. For further research, the mRNA and protein expression of integrin α_v_β_3_ and OPN were detected in HUVECs after TSH stimulation. Consistent with previous studies, the current results showed that the expression of integrin α_v_β_3_ increased in a dose-dependent manner with 0.1 to 10 mIU/mL TSH over 24 h at the same rate as OPN, which may indicate the ligand-receptor interaction of OPN and integrin α_v_β_3_ during the early stage of atherosclerosis.

After stimulation by specific factors, including proinflammatory cytokines and hormones, the activated endothelium expresses cell surface adhesion molecules such as VCAM-1 and intercellular adhesion molecule-1 (ICAM-1), which mediate interactions between the endothelial cells and circulating leukocytes [33,34]. Previous studies have shown that TSH may enhance the expression of ICAM-1 induced by TNF-α in a concentration-dependent manner [15]. In this work, another traditional endothelial adhesion molecule, VCAM-1, was detected. The results also showed more VCAM-1 expression in the aortic tissues of the SCH and CH groups than in the CON group.

Studies have proven that HUVECs can express TSHR, which is a g-protein-coupled cell surface protein receptor [35,36]. The TSHR signaling pathway is not only activated in thyroid epithelium, but is also functional in other cells, such as aorta cells [14], adipocytes [37], HUVECs [15], and fibrocytes [36]. It activates cAMP and the phosphoinositide 3-kinase (PI3K) pathway [38,39,40]. It then induces the activation of downstream kinases such as protein kinase C (PKC) and Akt (PKB). TSH treatment of the human dermal microvascular endothelial cell line (HMEC-1) was found to activate Akt and Erk1/2 [41], so levels of both kinases were measured in HUVECs. The results also showed the activation of p-Akt and p-Erk after TSH stimulation. However, the specific Akt inhibitor LY294002, but not the specific Erk inhibitor PD98059, stimulated levels of OPN, integrin α_v_β_3_, and VCAM-1, which might predict a proinflammatory role for Akt. Previous studies have shown that the PI3K/Akt signaling pathway plays a pivotal role in TNF-α induced activation of NF-κB signaling pathway; TSH was found to increase TNF-α levels in vivo and in vitro [42,43,44]. Consequently, it is feasible that TSH might play a role in the proatherosclerotic effect via the activation of Akt, independently or through TNF-α.

According to previous studies, TSH increased nitric oxide (NO), decreased vasoconstrictor endothelin, decreased tissue type plasminogen activator (t-PA) production, and had no effect on plasminogen activator inhibitor-1 (PAI-1) expression, which indicated that TSH had a protective effect on vasodilatation and induced the impairment of the fibrinolytic system in cultured human aortic endothelial cells [14]. Balzan et al. have demonstrated that TSH increased eNOS and vascular endothelial growth factor (VEGF) expression in human dermal microvascular endothelial cell line (HMEC-1), which might affect capillary network formation [41]. Tian et al. showed that TSH treatment in cultured HUVECs resulted in decreased eNOS and PGI_2_ expression and increased levels of endothelin-1 and PAI-1 compared to control [15]. Few articles show the relationship between TSH and endothelial function, but their conclusions are controversial. In the present study, our results showed that TSH increased the expression of OPN, VCAM-1, and integrin α_v_β_3_ in HUVECs, which were regarded as proatherosclerosis factors.

This study has some limitations. First, the animal experiments did not include female rats, which might limit the findings; second, the animal experiments might not have lasted long enough to show all the relevant morphological findings; Third, only HUVECs were used for the in vitro experiments. More types of endothelial cells might provide more useful results.

## 4. Materials and Methods

### 4.1. Materials

Human umbilical vein endothelial cells (HUVEC) and endothelial cell medium (ECM) were purchased from Sciencell Research Laboratories (Carlsbad, CA, USA). Thyrotropic hormone from bovine pituitary (T8931) and l-T_4_ were purchased from Sigma (St. Louis, MO, USA). Bovine TSH was prepared by dissolving 10mg of TSH in double distilled water to a final concentration of 1 IU/mL. Antibodies against osteopontin (ab63856) and integrin α_v_β_3_ (ab75872) were purchased from Abcam (Cambridge, UK). Antibodies against VCAM-1 (sc8304) were purchased from Santa Cruz Biotechnology (Dallas, TX, USA). Antibodies against GAPDH (TA-08), peroxidase-conjugated Affinipure goat anti-mouse IgG (H + L) (ZB-2305) and peroxidase-conjugated Affinipure goat anti-rabbit IgG (H + L) (ZB-2301) were purchased from Zhongshan Goldenbridge-Biotechnology (Beijing, China). Antibodies against phospho-p44/42 MAPK (Erk1/2) (Thr202/Tyr204) (4370S), and phospho-Akt (Thr308) (D25E6) (13038), PD98059 (9900), and LY294002 (9901) were purchased from Cell Signaling Technology (Danvers, MA, USA).

### 4.2. Animals

Thirty male Wistar rats weighing 180–200 g were purchased from Vital River (Beijing, China) and fed normal rat chow. The animal experiments were conducted according to the National Institute of Health Guide for the Care and Use of Laboratory Animals. All experiments were approved by the Animal Care and Use Committee of the First Affiliated Hospital of China Medical University (project identification code: 2010-I3, Date: 6 January 2010). The rats were randomly divided into three groups: subclinical hypothyroidism (SCH, *n* = 10), hypothyroidism (CH, *n* = 10), and control (CON, *n* = 10). The rats were injected with 10% chloral hydrate (i.p. 0.35 mL/100 g) and placed on an operating table. Rats in the CH and SCH groups were subjected to thyroidectomy, removal of the thyroid gland, to establish a hypothyroid model. Rats in the CON group were given a sham operation that did not involve removal of the thyroid gland, as described in a previous work [45]. Four weeks after surgery, the thyroidectomized rats of the SCH group were injected with l-T_4_ (s.c 1.0 µg/100 g) daily, and the rats in the CH and CON groups received injections of physiological sodium chloride solution. After surgery, all rats were provided with 0.1% (*w*/*v*) calcium lactate in their drinking water and fed normal rat chow.

### 4.3. Hormone Measurements

All blood samples from the three groups were immediately centrifuged at 13,000 rpm for 10min and stored at −80 °C for further research. Total T_4_ and TSH were detected using immunochemiluminometric assay (Immulite, Diagnostic Products Corporation, Los Angeles, CA, USA). The intra-assay coefficients of variation for TT_4_ and TSH were 4.34%–6.13% and 1.16%–4.12%, respectively. The inter-assay coefficients of variation for TT_4_ and TSH were 1.26%–3.27% and 1.73%–5.75%, respectively.

### 4.4. Cell Culture

HUVECs were maintained in ECM including basal medium, 5% fetal bovine serum, endothelial cell growth supplement, and penicillin/streptomycin solution, at 37 °C in a humidified incubator (95% air and 5% CO_2_). HUVECs were starved in serum-free ECM for 12 h before stimulation, and PD98059 and LY294002 were given 1 h before stimulation. The cells were then stimulated by recombinant bovine TSH at different concentrations and for different periods.

### 4.5. Western Blot

The aortas were removed from the rats, cut into several parts, and immediately stored at −80 °C for further use. HUVECs were stimulated by TSH (0, 0.1, 1, 10, 100 mIU/mL) for different times (0, 2, 6, 12, 24, 48 h), or pretreated with 20 µmol/L PD98059 or 20 µmol/L LY294002, washed with PBS, and incubated in lysis buffer for 30 min on ice. All protein samples (30 µg for cells, 50 µg for tissues) were separated by 10% SDS-acrylamide gel electrophoresis and transferred onto PVDF membranes (Millipore, Billerica, MA, USA) at a constant voltage of 100 V for 100 min. Then, the membranes were incubated at 4 °C overnight with the following primary antibodies: at anti-osteopontin (ab63856,1:500), anti-integrin β 3 (ab75872, 1:500), anti-VCAM-1 (sc8304, 1:100), anti-GAPDH (TA-08,1:5000), anti-phospho-p44/42 MAPK (Erk1/2) (Thr202/Tyr204) (4370S, 1:1000), anti-p44/42 MAPK (Erk1/2) (4695S, 1:1000), anti-Akt (pan) (C67E7) (4691, 1:1000), anti-phospho-Akt (Thr308) (D25E6) (13038, 1:1000). After that, the membranes were incubated for 2 h with the following secondary antibodies: peroxidase-conjugated Affinipure goat anti-mouse IgG (H + L) (ZB-2305, 1:5000) and peroxidase-conjugated Affinipure goat anti-rabbit IgG (H + L) (ZB-2301, 1:2000). Bands were detected by chemiluminescence using an ECL detection system. The relative densitometry of the band was measured using Image J software (Bethesda, MD, USA).

### 4.6. Quantitative Real-Time PCR

Total RNA was used for RNA extraction using TRIzol reagent according to the manufacturer’s instructions. Then RNA was reverse-transcribed to cDNA using a TaKaRa PrimeScript RT Master Mix (Takara, Shiga, Japan) and amplified in a total volume of 20 µL. The samples were mixed with SYBR Premix Ex TaqII, primers, and DEPC water. PCR was performed for 40 cycles with an initial denaturing step at 95 °C for 5 s and annealing at 60 °C for 30 s and were terminated by a cooling step at 50 °C using a LightCycler Real-Time PCR System (Roche 480, Berlin, Germany). The primers were synthesized by TaKaRa. They are presented in Table 2.

### 4.7. Immunohistochemistry (IHC)

The aorta of each rat was washed in PBS and fixed in 4% paraformaldehyde (Beijing Chemical Works, Beijing, China) for 24 h. Paraffin sections were cut to sections 4 µm thick for immunohistochemical staining. The tissue sections were dewaxed and hydrated (Mai Xin_Bio, Fuzhou, China), then boiled to restore antigen epitopes and incubated overnight at 4 °C with the following antibodies: anti-osteopontin (ab63856, 1:50), anti-integrin β_3_ (ab75872, 1:50), and anti-VCAM-1 (sc8304, 1:50). Then, the sections were washed with PBS and incubated with anti-rabbit or anti-mouse IgG-HRP antibody (Boster, Wuhan, China) for 30 min at 37 °C. The sections began to turn brown after approximately 5–10 min of exposure to 3,3′-diaminobenzidine (DAB) (Mai Xin Bio) for different antibodies. To assess nonspecific staining, some sections were incubated without primary antibodies. Five visual fields per slide were randomly selected. To assess the expression of each target, the optical density (OD) of positive cells in each field was calculated using a MetaMorph/DPIO/BX51 morphology image analysis system (Olympus, Tokyo, Japan), and the results were analyzed using ImagePro Plus software (Bethesda, MD, USA).

### 4.8. Transmission Electron Microscope (TEM)

Each group of aorta samples was dissected into pieces and fixed with 2.5% glutaraldehyde (Sinopharm Chemical Reagent Co., Ltd., Shanghai, China) at 4 °C. After stimulation, HUVECs were washed with PBS and harvested by trypsinization, centrifuged at 1000 rpm for 5 min and fixed with 2.5% glutaraldehyde at 4 °C for 24 h. Then the samples were post-fixed with 1% osmium tetroxide at 4 °C (1 h for HUVECs, 2 h for aorta tissue) and washed with PBS three times. Specimens were dehydrated in ethanol (Beijing Chemical Works, Beijing, China) with a gradient series and 100% acetone (Beijing Chemical Works), infused with Epon812 (Serva, New York, NY, USA) and embedded in pure Epon812 at 65 °C (48 h for HUVECs, 72 h for aorta tissue). After the semi-thin sections were observed, the aortic cross-section was located, and five serial ultra-thin sections (70 nm) from each rat were stained with 4% uranyl acetate (Kojima Chemicals Co., Ltd., Tokyo, Japan) and lead citrate (Alfa Aesar, Ward Hill, MA, USA). The samples were examined with TEM (H-7650, Hitachi, Tokyo, Japan).

### 4.9. Statistical Analysis

All results were expressed as the means ± SEM. Comparisons between groups were examined by one-way ANOVA followed by Student-Newman-Keuls testing using SPSS 20.0 software (SPSS, Chicago, IL, USA). *p*-value < 0.05 was considered statistically significant. All graphs were constructed using Graph Prism (5.0) software (San Diego, CA, USA).

## 5. Conclusions

In conclusion, rats with subclinical hypothyroidism showed high levels of OPN, integrin α_v_β_3_, and VCAM-1 expression in their aortic tissues. TSH induced OPN, integrin α_v_β_3_, and VCAM-1 expression in HUVECs. This process involved the activation of Akt. These proatherosclerosis factors may become new biomarkers for the early diagnosis of atherosclerosis in subclinical hypothyroidism patients.

## Figures and Tables

**Table 1:** Plasma levels of TT4 and TSH in each group.

	CON		SCH		CH	
	TSH (mIU/L)	TT_4_ (μg/dL)	TSH (mIU/L)	TT_4_ (μg/dL)	TSH (mIU/L)	TT_4_ (μg/dL)
4 Weeks after surgery	0.19 ± 0.10	4.24 ± 0.82	21.65 ± 8.54 *	1.52 ± 0.93 *	21.8 ± 3.65 *	1.34 ± 0.35 *
4 Weeks after l-T_4_ injection	0.36 ± 0.19	4.01 ± 0.97	4.01 ± 0.97	3.16 ± 1.09	13.0 ± 6.09 *	1.61 ± 0.55 *
14 Weeks after l-T_4_ injection	0.37 ± 0.26	3.51 ± 0.97	6.79 ± 3.70 *	3.86 ± 0.78	16.14 ± 4.58 *	1.77 ± 0.73 *

Values are presented as the means ± SEM; * *p* < 0.05, versus CON group; SEM, standard error of the mean; TSH, thyrotropin; TT_4_, total thyroxine.

**Table 2:** Sequence of primers used for Real Time-PCR.

Gene	Primer (5′ to 3′)
*VCAM1*	Forward: GCGGAGACAGGAGACACAGTACTAA
	Reverse: GAGCACGAGAAGCTCAGGAGAA
*SPP1*	Forward: ACAGCCACAAGCAGTCCAGATTA
	Reverse: TCCTGACTATCAATCACATCGGAAT
*ITGB3*	Forward: GAGGTCATCCCTGGCCTCAA
	Reverse: CTGGCAGGCACAGTCACAATC
*GAPDH*	Forward: CAATGACCCCTTCATTGACC
	Reverse: GACAAGCTTCCCGTTCTCAG
